# FEM Analysis of Mandibular Prosthetic Overdenture Supported by Dental Implants: Evaluation of Different Retention Methods

**DOI:** 10.1155/2015/943839

**Published:** 2015-12-21

**Authors:** M. Cicciù, G. Cervino, E. Bramanti, F. Lauritano, G. Lo Gudice, L. Scappaticci, A. Rapparini, E. Guglielmino, G. Risitano

**Affiliations:** ^1^Human Pathology Department, University of Messina, Messina, Italy; ^2^Medical Sciences and Odontostomatology Department, University of Messina, Messina, Italy; ^3^Mechanics and Infrastructures Department, “Guglielmo Marconi” University of Rome, Rome, Italy; ^4^Department of Electronic Engineering, Chemistry and Industrial Engineering, University of Messina, Messina, Italy

## Abstract

Prosthetic rehabilitation of total edentulous jaws patients is today a common technique that clinicians approach in their daily practice. The use of dental implants for replacing missing teeth is going to be a safe technique and the implant-prosthetic materials give the possibility of having long-term clinical success. Aim of this work is to evaluate the mechanical features of three different prosthetic retention systems. By applying engineering systems of investigations like FEM and von Mises analyses, how the dental implant material holds out against the masticatory strength during the chewing cycles has been investigated. Three common dental implant overdenture retention systems have been investigated. The ball attachment system, the locator system, and the common dental abutment have been processed by Ansys Workbench 15.0 and underwent FEM and von Mises investigations. The elastic features of the materials used in the study have been taken from recent literature data. Results revealed different response for both types of device, although locator system showed better results for all conditions of loading. The data of this virtual model show all the features of different prosthetic retention systems under the masticatory load. Clinicians should find the better prosthetic solution related to the patients clinical condition in order to obtain long-term results.

## 1. Introduction

Many edentulous patients have difficulty in functioning, speaking, chewing, and eating, leading to a decline in their quality of life. This is especially evident in the subjects having an edentulous mandible where partial or total prosthesis cannot give patients aesthetics and function. This particular condition is often due to the lack of the inferior total prosthesis stability. In the upper jaw, the total prosthesis finds its stability and function by the possibility of using the palate surface; in the lower jaw, the presence of the tongue reduces the area of the prosthesis and consequently the instability of the prosthesis increases. Nowadays, the possibility of positioning two or more dental implants in the anterior mandible allows methods of additional retention to be used to stabilize partial or complete lower dentures [1–5].

Quality assurance of health care delivery has emphasized the importance of patient's perceptions of medical interventions and treatments since 1970s. The patient expectations before and after a treatment are crucial to the final satisfaction from the treatment outcomes and this is today a continuing challenge for clinicians and researchers [6–9]. This is even more critical today, as the current practice of Evidence Based Medicine requires that the patients are actively engaged in the decision making with regard to their treatment. In addition, understanding and measuring the expectations of patients prior to treatment appear to be an essential prerequisite to have final successful patient reported long-term clinical outcomes [10, 11].

Implant-retained overdentures have become an important treatment option of modern dentistry. Such treatment presents the opportunity of high levels of oral health related quality of life and is particularly important in terms of population aging as edentulousness percentage continues to be relevantly high [12]. For mandibular implant-based overdentures, current consensus is that patients' satisfaction and quality of life are significantly greater for implant-supported overdentures than for conventional dentures and that a two-implant mandibular overdenture should be the minimum treatment standard for most patients giving a social possibility of low cost therapies. Not least, the availability of evidence already facilitated an assessment of the cost-effectiveness of implant-retained mandibular overdentures [1, 13–15].

Implant-retained mandibular overdenture presents a reliable and simple solution to denture retention and stability. The retention and stability characteristics are provided mainly by implants through attachments. So, various types of attachment systems have been proposed for connecting implant-retained mandibular overdentures to underlying implants. Independent connections to each implant abutment with O-rings, or splinting of implants with bar/clip attachments, are the most common approaches that have been used. Bar overdenture is a popular choice because of its load sharing [16–18].

To our knowledge, the effectiveness of mandibular overdentures retention systems has never been investigated before. Therefore, the purpose of the present study was to check the value of three different retention systems by FEM and von Mises analysis in order to underline which system offers best efficiency on the long-term control. Since there is no other similar study evaluating stress generated on different attachment types over vertical and angular strength, the purpose of this study is also to consider the stress distribution around implants, bone, and prosthetic component by 3D models.

## 2. Material and Methods

The investigation was performed on single tooth dental implant and prosthetic elements of retention in order to point out possible failure related to the fracture of structural components or to overload on bone tissue. Three different systems of retention have been recorded and evaluated over vertical and angular stresses. The FEM assists the field of oral implantology also to understand the characteristics of each implant-prosthetic component, its physicochemical and optimal environmental conditions, and its close relationship with hard and soft tissues. The dissipation of the tensional forces depends not only on the number and distribution of the positioned implants, but also on the structure material and the shape and size of the individual components of implant-prosthetic structure.

With the finite element analysis, it was demonstrated that even a small change in the shape of the abutment implant could influence the distribution of the stress on the structures of prosthetic and biological ones. So the FEM also gives directions to the operator on the most favourable choice of the type of implant and the type of prosthesis, compared to a predetermined clinical image.

The main difficulty in simulating the behaviour of compensatory biomechanical bone-implant prosthesis compared to the forces of tension lies in the modelling of the maxilla and mandible jaws and their reaction to the masticatory load [13, 16].

Some key factors that influence the accuracy of the results of the FEM should be considered. Among these are

- detailed geometry of the system and the surrounding bone to be modelled,
- boundary conditions and constraints,
- material properties,
- load conditions,
- bone-implant interface,
- test of convergence,
- validation of the model.

The geometry of the virtual model finite element should be the clinical reality as precisely as possible to get results biomechanical plausible. Solid models of the jaw arches, the dental implants, and the prosthetic crowns are constructed from CT images, which are processed through a CAD (computer-aided design) in 3D FEM. The informatics programs to recreate the virtual three-dimensional CAD model were used: during the reverse engineering Corel Draw was used which is powerful vector graphics software, through which it was possible to rebuild and scale sections of the facility object of study. The reconstruction of three-dimensional model was performed in Ansys Workbench, using the vector geometries output from Corel Draw. The analysis process was then divided into two phases: preprocessing, construction phase of the finite element model, and postprocessing, processing and representation of the solutions [1, 8, 13, 18].

### 2.1. Reverse Engineering and CAD Model

**Figure 1:**
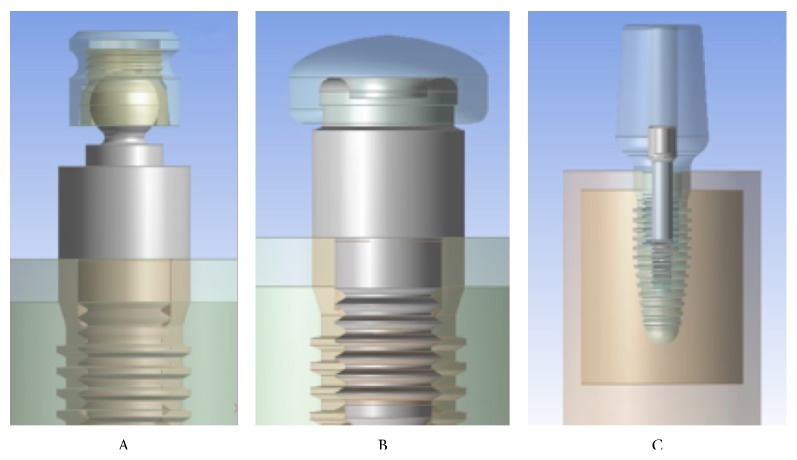
Example of the three models of the prosthetic retention systems. Study A, ball attachment; study B, locator; study C, universal abutment.

**Figure 2:**
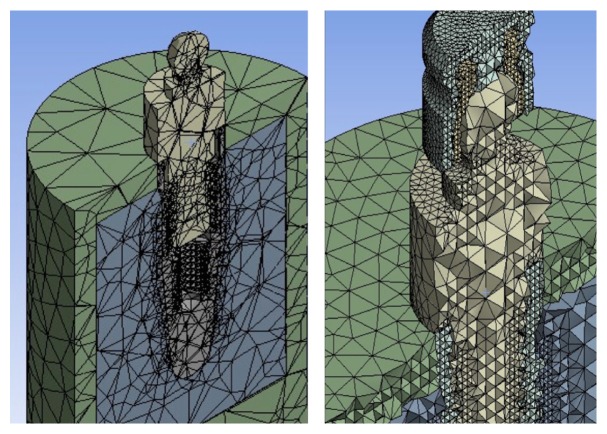
Example of standard mesh automatically generated mesh and final. The greater accuracy of the elements in the second case can be noted.

In our created models, the dental implant with all its components was recreated by using Corel Draw Graphic Suite X7 (Figures 1 and 2).

The main dimensions are deducted from the implant-prosthetic components and made real by the small details of their physicochemical characteristics provided by the scientific literature and catalogues. The modelling phase, by which the information passed from the physical system to a mathematical model, is composed of extrapolating from the same number of variables and “filtering out” the remaining ones. It was performed in Ansys Workbench. Where implant-prosthetic threads are present, helicoids were properly reshaped.

### 2.2. The Finite Element Analysis

Then, after obtaining these models' three-dimensional CAD, the FEA jaw-implant-prosthesis was performed with Ansys Workbench 15.0, program characterized by a bidirectional connectivity CAD, by high productivity, and by an innovative design vision that binds the entire simulation process.

A 3D linear static structural simulation was performed showing the relation (stress and strain) between bone and implant prosthodontics elements: ball attachment, locator, and common screwed abutment.

### 2.3. Choice of Materials

In this experimental study, we chose titanium grade 4 for the construction of the dental fixture, the abutment screw, abutment, and all the prosthetics components.

The properties of materials have been specified in terms of Young's modulus and Poisson's ratio and density. The different physical behaviour of materials with respect to the loading forces has been considered.

In our case, the alloys of titanium have a plastic behaviour, thanks to which are resistant. Titanium is able to absorb loads, even intense ones, possibly meeting a permanent deformation but without tending to fracture. Moreover, titanium alloys have a limit of resistance at least 5 times greater than that of the ceramic, can be subjected to a voltage of up to 1000 MPa (equivalent to 1000 kg on each mm^2^ of surface), and do not involve rupture of the crash, or fractures per pulse. For this reason, in our 3D model, the more resistant component within the implant-prosthesis system is precisely the dental implant [1, 12, 15].

The bones structure was considered orthotropic (for both the cortical and the cancellous bone); the reference values were taken from the literature [1–6].  Table 1 shows the characteristics of resistance and elasticity, which are considered for each component.

### 2.4. Creating the Correct Mesh

**Figure 3:**
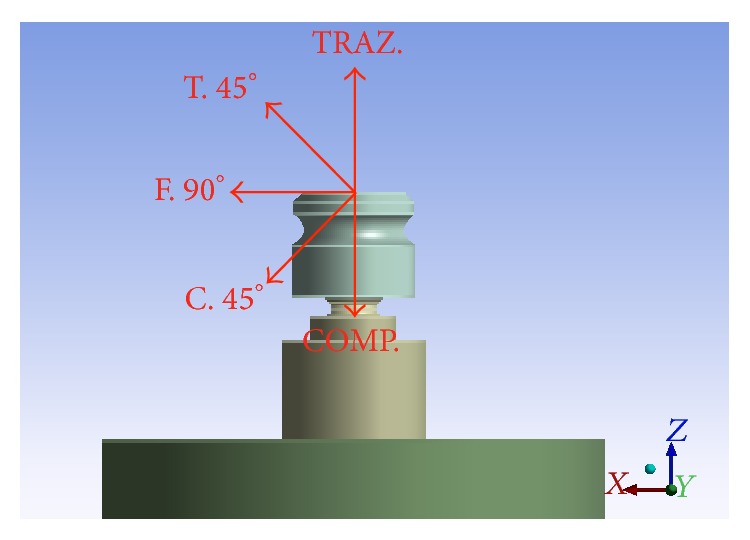
Different load type used in the studies.

The discretization of the geometry has the aim to obtain a discrete model of a continuous object and consisting in a finite number of freedom degrees (meshing). A polygonal mesh is a collection of vertices, edges, and faces that defines the shape of a polyhedral object in 3D computer graphics and solid modelling (Figures 1, 2, and 3).

The basic unit of a mesh is the voxels (volume pixels): in geometric modelling, the volume that contains the object for modelling it in a three-dimensional grid of positions is divided. Each position determines the presence or absence of material. The more it gets closer to the areas of interest, the more the mesh increases in number; clearly, a condensation of the mesh results in a dilation of the calculation time. Discretization was performed using elements SOLID186 and SOLID187 of the Ansys library.

SOLID186 is a higher order 3D 20-node solid element that exhibits quadratic displacement behaviour, while SOLID187 element is a higher order 3D, 10-node element. Both of them well suit modelling irregular meshes. The patch independent algorithm was used to better model the inner side of the model and the minimum size of the elements was set to 0,2 mm. In this way, it was possible to represent all the threads with a very good quality. A maximum skewness of about 0,8, with a standard deviation of about 0,16 for all the models, is an idea of a good quality mesh.

Mesh details are as follows:

 Model A: 1.030.584 nodes and 711.404 elements.
 Model B: 1.058.506 nodes and 737.849 elements.
 Model C: 1.016.490 nodes and 703.098 elements.

### 2.5. Loading Conditions

The three implants were tested with static loads. Different loading conditions were considered:

- Pure traction of 400 N.
- Pure compression of 400 N.
- Flexural force of 400 N.
- Mixed tensile-bending of 400 N.
- Mixed compression bending of 400 N.

All loads were distributed on the implant surface in contact with the tooth.

### 2.6. Constraint Conditions and Contacts

Since the activity in question concerns the comparison of most types of plant subject to the same load conditions, it was considered appropriate to be used as stationary on the ground (fixed) of the outer side surface of the models. All the contacts modelled in this study are considered linear. The bone-implant and the bone-bone contact conditions established in this FEM analysis are considered bonded. In this research, the perfect osseointegration with total contact surface between the implant and the bone was simulated, with no possibility scroll between the two areas. Moreover, for all the threaded connections was considered a bolt pretension in accordance with the installation requirements. In particular, implant/bone = 46,5 N, abutment/implant = 50 N, and bolt/implant = 62,5 N (Table 1).

The following elements have been selected and evaluated:

 Dental implant “Root Line 2” cod. K1032.4313 (Ø4,3 × 13 mm),* titanium grade 4*.
 Abutment “ball” cod. J2249.4330 (Ø4,3 A.G.3 mm),* titanium grade 4*.
 Abutment “locator” cod. J2253.4330 (Ø4,3 A.G.3 mm),* titanium grade 4*.
 “Universal” abutment cod. K2211.4300 (Ø4,3 H11 mm),* titanium grade 4*.
 Screw cod. J4005.1601 (M1,6),* titanium grade 4*.
 “Bone simulated tissue,”* cortical and cancellous bone*.

The three systems evaluated have been classified as follows.

Model A is as follows:

- Dental implant “Root Line 2” cod. K1032.4313 (Ø4,3 × 13 mm).
- Abutment “Ball” cod. J2249.4330 (Ø4,3; gingival height 3 mm).
- Bone (midollar and cortical).

Model B is as follows:

- Dental implant “Root Line 2” cod. K1032.4313 (Ø4,3 × 13 mm).
- Abutment “locator” cod. J2253.4330 (Ø4,3; gingival height 3 mm).
- Bone (midollar and cortical).

Model C is as follows:

- Dental implant “Root Line 2” cod. K1032.4313 (Ø4,3 × 13 mm).
- “Universal” abutment cod. K2211.4300 (Ø4,3 H11 mm).
- Passing screw cod. J4005.1601 (M1,6).
- Bone (midollar and cortical).

Authors decided to choose a general grid with a different method for performing the paramtric evaluation. All elements of the geometry have been assigned a discretization method provided for the use of tetrahedral shape with independent algorithm and with a lower limit of size 0,2 mm (method: tetrahedrons, independent patch, min. size 0,2 mm).

These settings have met the needs and it is noticed that in the areas of interest of the threads the discretization was very fine. This required, however, very hard work by the microprocessor of the computer, because the final grid contains more then one million nodes.

The following were thus obtained: study A, 1,030,584 knots and 711,404 items; study B: 1,058,506 knots and 737,849 items; and study C: 1,016,490 knots and 703,098 items (Figures 1, 2, and 3).

In Figures 1, 2, and 3 are some examples of the model discretized; you may notice the difference between the mesh automatically created and the one used for testing as the size of the elements is considerably reduced in the vicinity of the zones of interest.

It then passes to the definition of the forces in play. Several studies have been done comparing implants loaded vertically and angled. Authors found that the inclination of the implants greatly influences the stress concentration around them [1, 17, 19]. In the literature, the value of loads is very variable; Michailidis et al. [2] chose a load of 400 N equal to what was said by Borchers and Reichart [22] so that the value does not exceed the value of risk for the bone (450 N) as specified in Section 2. All the 3 studies were subjected to different types of static load:

 Compression pure (COMP.) = 400 N (along *Z*).
 Compression 45° (45°C) = 400 N (282.843 along *X*+ 282.843 along *Z*−).
 Bending pure (90°F) = 400 N (along *X*+).
 Traction A 45° (45°T) = 400 N (282.843 along *X*+ 282.843 along *Z*+).
 Pure drive (TR.) = 400 N (along *Z*+).

The loads, as can be noted, correspond to those obtained on the molar area for extreme operating, reported in the literature [1, 3, 18–22]. The other types of cargo are mixed and being angled with respect to the symmetry axis *Z* that will generate a bending moment in the system. The influences of the soft tissues on the neighbourhood of the pillars (3 mm on thickness) are considered to be negligible, and also all the threads have been considered horizontal and not helical.

In Model A, the forces are applied on the upper surface and side of the matrix of retention because through this the forces, crossing first the prosthesis, then the matrix of retention, the pillar, and the facility, are downloaded bone. In Model B, forces are placed on top and side of the insert retention, as is this cap, which acts as an intermediary between the prosthesis and the implant. In Model C, forces are applied on the upper surface and side of the projection apical abutment, because it will be the “internal skeleton” to the final prosthesis and will therefore be along its entire apical surface upper solicited. Then, we need to define constraints. In the systems under study, a constraint on the base and interlocking on the exterior surface of the cylinder of bone has been imposed so as to make the system similar to reality (Figures 2 and 3).

The last step is to define what kind of solutions we are interested in obtaining, in order to have comparable results with past studies already carried out on other types of the dental implant. The theory used to determine the stress distribution of the various investigations is that the von Mises in the specific within the simulation program is called equivalent stress.

## 3. Simulations and Results

Simulations have required intensive use of the computer processor, with timing of response ranging from 20 to 30 minutes per simulation. The results obtained with the simulation demonstrate the relationship between the loads applied to the system, the geometrical characteristics of the materials, the constraints, and deformations. One of the theories most used to determine the stress is von Mises, already described in Section 2.6. This theory has been applied to this experimentation to determine the stress distribution of the various studies. The program expresses the results in the form of a chromatic scale of colors ranging from blue to red for the minimum values and for the maximum values. The values represent those of the respective solution found. The values found were compared with the critical values of the respective materials:

 Titanium grade 4 (arrangement/insert): *σs* = 485 MPa, *sr* = 550 MPa.
 Titanium alloy (pillars/abutment/screw): *σs* = 830 MPa, *sr* = 900 MPa.
 Cortical bone: compression *sr* = 182 MPa, *sr* = 195 MPa.
 Tensile *σs* = 155 MPa, *sr* = 133 MPa.
 Cancellous bone: compression *σs* = 51 MPa, *sr* = 55 MPa.
 Tensile *σs* = 32.4 MPa, *sr* = 37.5 MPa.
 Elitor (sheath): *σs* = 700 MPa, *sr* = 855 MPa.

Data are collected in the table for each model. The following images that highlight the achievements are reported also. For every one of the 5 tests will be reported 2 photos of the elastic deformation equivalent von Mises (the whole and especially the area of maximum value) and two photographs of the voltage equivalent von Mises (the whole and especially the area of maximum value).

The values that do not fit these parameters are italic when the voltage exceeds the yield point and bold when the voltage exceeds the breaking point.

The areas of maximum voltage in the individual components are as follows:

- Insert: last thread bottom in contact with the inner sheath.
- Inner sheath: lower area of contact with the sphere pillar.
- Pillar: upper neck pre sphere.
- Plant: outer upper surface in contact with pillar.
- Cortical bone: surface between implant neck top and cancellous bone.
- Cancellous bone: last thread with facility.

**Figure 4:**
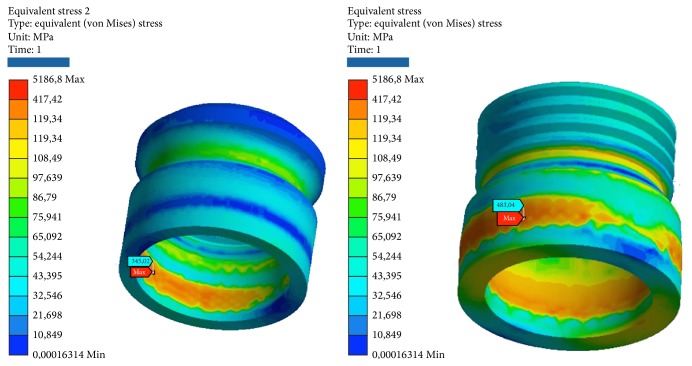
Areas of maximum tension for the two components (internal and external) of the matrix of retention Dalbo CM-Plus bending pure stress.

**Figure 5:**
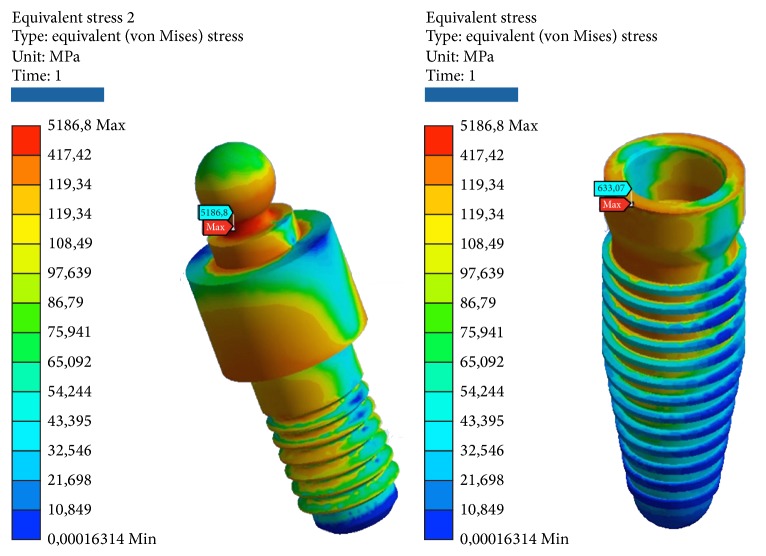
Areas of maximum voltage for the pillar and the implant bending pure stress.

**Figure 6:**
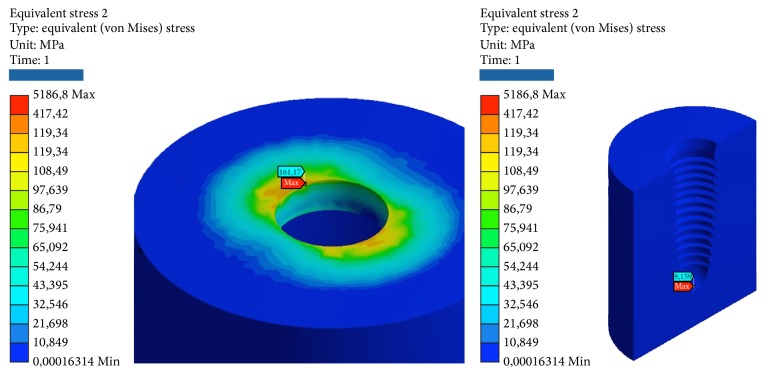
Areas of greatest tension for the cortical and cancellous bone under bending pure stress.

*Model A.* At compression, the maximum stresses are as follows (Figures 4, 5, and 6):

- Matrix retention Dalbo CM-Plus (formed by insert outer and inner sheath): very low, far below the critical values.
- Ball abutment: high, more than half of the critical values in the area at the upper narrow section before the ball connection with the matrix. Although the values are high, we are still far below the values that cause yielding or breakage. The lower area of the pillar coupled by means of threading the implant resists the load well.
- System: low, far below the critical values.
- Cortical bone: average, about half of the critical values.
- Cancellous bone: low, far below the critical values.

At compression/bending of 45°, maximum stresses are as follows:

- Matrix retention Dalbo CM-Plus (formed by insert external and inner sheath): average, about half of the critical values.
- Ball abutment: very critical, beyond the limits of rupture in the area in the upper narrow section of the sphere before connection with the matrix. It follows the failure of the component. In this area, we also have the maximum elastic deformation. The lower area of the pillar coupled by means of threading the implant resists the load instead.
- Implant: very critical, beyond the limits of rupture, even for a few MPa. It follows the failure of the component.
- Cortical bone: high, more than half of the critical values.
- Cancellous bone: low, far below the critical values.

At decrease of 90°, we have the worst conditions. The maximum stresses are as follows:

- Matrix retention Dalbo CM-Plus (formed by insert external and inner sheath): average, about half of the critical values.
- Ball abutment: very critical, beyond the limits of rupture. It results in the break in the area of the component to the neck of the bottle before the top spherical. In this area, we also have the maximum elastic deformation. The lower area of the pillar coupled by means of threading the implant resists the load instead.
- System: very critical, beyond the limits of rupture. It follows the failure of the component.
- Cortical bone: very critical, beyond the limits of rupture.
- Cancellous bone: very low, far below the critical values.

At tension/flexion of 45°, we find almost the same values of the maximum stresses that we had at compression/flexion of 45°:

- Matrix retention Dalbo CM-Plus (formed by insert external and inner sheath): average, about half of the critical values.
- Ball abutment: very critical, beyond the limits of rupture in the area in the upper narrow section of the sphere before connection with the matrix. It follows the failure of the component. In this area, we also have the maximum elastic deformation. The lower area of the pillar coupled by means of threading the implant resists the load instead.
- Implant: very critical, beyond the limits of rupture, even for a few MPa. It follows the failure of the component.
- Cortical bone: very critical, beyond the limits of rupture.
- Cancellous bone: low, far below the critical values.

At traction, we recorded voltage values similar to those seen for compression. The maximum stresses are as follows:

- Matrix retention Dalbo CM-Plus (formed by insert outer and inner sheath): very low, far below the critical values.
- Ball abutment: high, more than half of the critical values in the area at the upper narrow section before the ball connection with the matrix. Although the values are high, we are still far below the values that cause yielding or breakage. The lower area of the pillar coupled by means of threading the implant resists the load well.
- System: low, far below the critical values.
- Cortical bone: average, about half of the critical values.
- Cancellous bone: low, far below the critical values (Tables 2 and 3).

**Figure 7:**
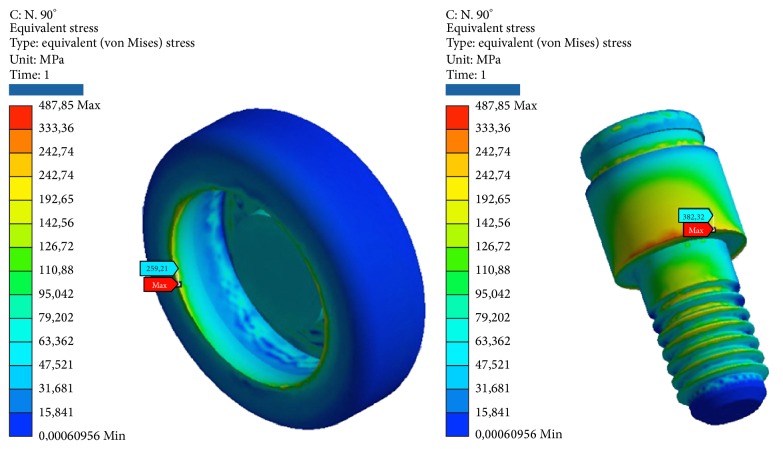
Areas of maximum voltage for the retention insert and the pillar locator bending pure stress.

**Figure 8:**
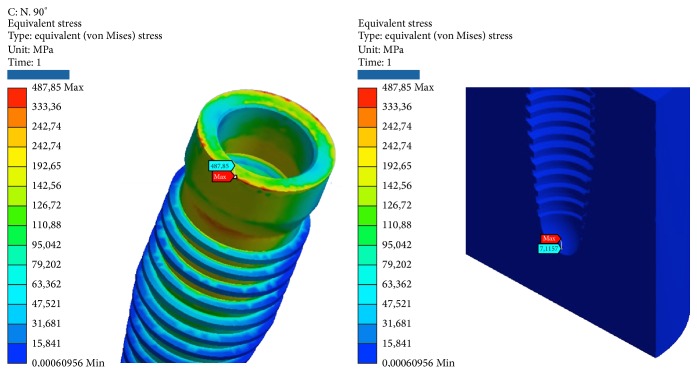
Areas of maximum stress for the implant and the cancellous bone under bending pure stress.

**Figure 9:**
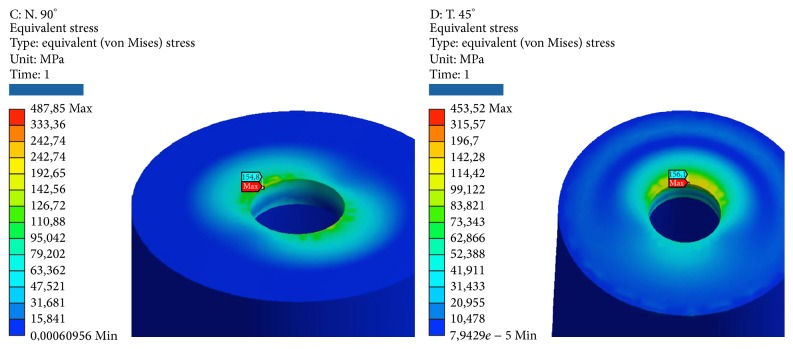
Areas of maximum voltage for the cortical bone to pure bending and tensile at 45°.

*Model B.* At compression, the maximum stresses are as follows (Figures 7, 8, and 9):

- Retention insert: low, well below the critical values.
- Pillar locator: very low, far below the critical values. The lower area of the pillar coupled by means of threading the implant resists the load well.
- Implant: average, about half of the critical values.
- Cortical bone: low, far below the critical values.
- Cancellous bone: very low, far below the critical values. At the apex, we find the maximum value of elastic deformation.

At compression/bending of 45°, the maximum stresses are as follows:

- Pillar locator: low, far below the critical values. The lower area of the pillar coupled by means of threading the implant resists the load well.
- Implant: tall, next to the critical values.
- Cortical bone: high, more than half of the critical values. Although the values are high, we are still below the values that cause yielding or rupture in compression, which, it must be remembered is much higher for this material compared to the values of traction.
- Cancellous bone: very low, far below the critical values. At the apex, we find the maximum value of elastic deformation.

At decrease of 90°, we have the worst conditions. The maximum stresses are as follows:

- Retention insert: average, about half of the critical values.
- Pillar locator: average, about half of the critical values. The value is still only half of those critical values that would cause rupture. The lower area of the pillar coupled by means of threading the implant resists the load well.
- System: critical, very little beyond the limits of yield. It follows the yield component.
- Cortical bone: criticism, beyond the limits of yield.
- Cancellous bone: very low, far below the critical values. At the apex, we find the maximum value of elastic deformation.

At tension/flexion of 45°, we find almost the same values of the maximum stresses that we had at compression/flexion of 45°:

- Insert retention: low, far below the critical values.
- Pillar locator: low, far below the critical values. The lower area of the pillar coupled by means of threading the implant resists the load well.
- Implant: tall, next to the critical values.
- Cortical bone: very critical, beyond the limits of rupture. It follows the breakdown of bone. It should be remembered that the bone characteristics can vary from individual to individual and that the limit values of yield and breaking are only estimates.
- Cancellous bone: very low, far below the critical values. At the apex, we find the maximum value of elastic deformation.

At traction, we recorded voltage values similar to those seen for compression. The maximum stresses are the following:

- Insert retention: low, far below the critical values.
- Pillar locator: very low, far below the critical values. The lower area of the pillar coupled by means of threading the implant resists the load well.
- Implant: average, about half of the critical values.
- Cortical bone: low, far below the critical values.
- Cancellous bone: very low, far below the critical values. At the apex, we find the maximum value of elastic deformation (Tables 4 and 5).

*Model C.* At compression, the maximum stresses are the following:

- Abutment: outer edge in contact with the implant and the cortical bone.
- Screw connection: in the tests on the angled outer edge on the head in contact with the abutment, in the tests of compression/tensile, as expected, tensions scarcano more in the throat near the first thread that is screwed to the system.
- Implant: upper outer edge in contact with the abutment.
- Cortical bone: upper edge with facility.
- Cancellous bone: in the apex, in the area in contact with the last thread on the bottom of the implant and for testing angle.

At compression/bending of 45°, the maximum stresses are the following:

- Universal abutments: more than half of the critical values. Although the values are high, we are still far below the values that cause yielding or breakage.
- The connection screw: low, well below the critical values.
- System: very critical, beyond the limits of rupture. It follows the failure of the component.
- Cortical bone: high, more than half of the critical values. Although the values are high, we are still below the values that cause yielding or breakage.
- Cancellous bone: low, far below the critical values. At the apex, we find the maximum value of elastic deformation.

At decrease of 90°, we have the worst conditions. The maximum stresses are the following:

- Universal abutment: high, more than half of the critical values. Although the values are high, we are still below the values that cause yielding or breakage.
- The connection screw: medium, about half of the critical values.
- System: very critical, beyond the limits of rupture. It follows the failure of the component.
- Cortical bone: very critical, beyond the limits of rupture. It follows the failure of the component. It should be remembered that the bone characteristics can vary and no limit values are only indicative yield.
- Cancellous bone: low, far below the critical values. At the apex, we find the maximum value of elastic deformation.

At tension/flexion of 45°, we find almost the same values of the maximum stresses that we had at compression/flexion of 45°:

- Universal abutment: high, more than half of the critical values.
- The connection screw: low, well below the critical values.
- System: very critical, beyond the limits of rupture. It follows the failure of the component.
- Cortical bone: very critical, beyond the limits of rupture. It follows the failure of the component. It should be remembered that the characteristics and values of bone can vary and the breaking points are only estimates.
- Cancellous bone: low, far below the critical values. In the last thread of the system, we find the maximum value of elastic deformation.

**Figure 10:**
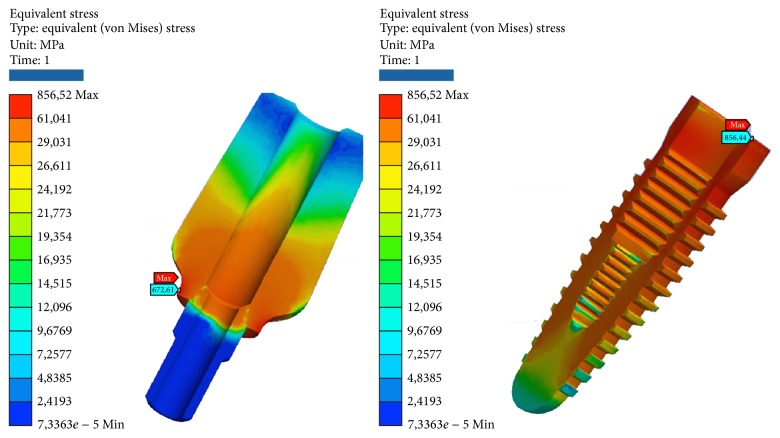
Areas of maximum voltage for the abutment and the fixture.

**Figure 11:**
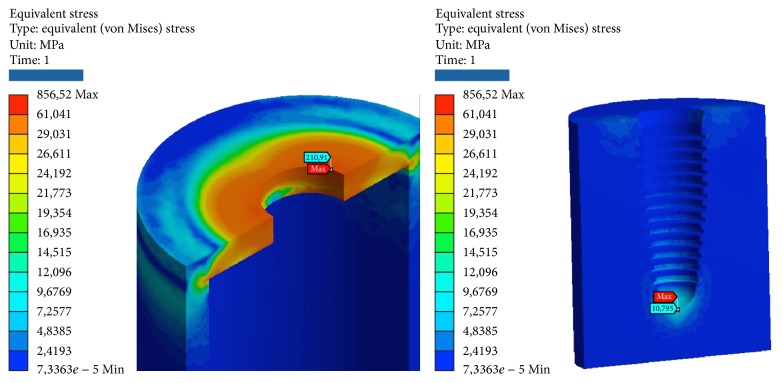
Areas of maximum voltage for the cortical bone and midollar bone at pure flection.

**Figure 12:**
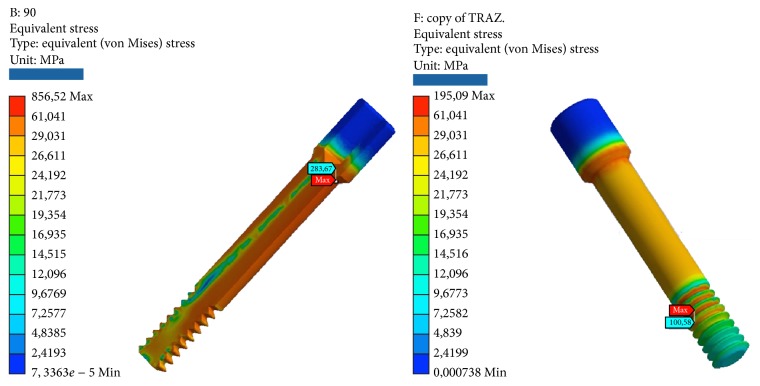
Areas of maximum voltage for the passing screw for flection and traction.

At traction, we recorded voltage values similar to those seen for compression. The maximum stresses are the following:

- Universal abutments: low, far below the critical values.
- The connection screw: very low, far below the critical values. The lower zone coupled by means of threading the implant resists the pressure load.
- Implant: average, about half of the critical values.
- Cortical bone: average, about half of the critical values.
- Cancellous bone: very low, far below the critical values. At the apex, we find the maximum value of elastic deformation (Tables 6 and 7; Figures 10, 11, and 12).

## 4. Discussion

Stress distribution around dental implants or the peri-implant bone tissue is a quite debated topic in the recent literature [1, 4, 7, 20–27]. However, clinicians and researchers aimed to record the long-term efficiency of those biomedical materials in order to guarantee stable long-term clinical results. Talking about the overdenture placed over dental implants, the main problems related to the material seem to be connected with the material composition and with the fatigue during the masticatory cycles.

Model A, formed by the ball abutment, highlighted some problematic issues with the neck of the upper connection with the spherical part. In this area, because of the reduced section, it has a large stress concentration that leads, in the cases of loads very angled, to breakage. The advantage of having a degree of freedom in the more you “pay dearly” and care should be taken in using this component. Only in cases of pure tension and compression system it has withstood the strain. The lower coupling, with metric thread M3 (of size relatively larger than the screw of the study all'M1,6 C), has instead to be great, and because it allows large accumulations of tension, because it is being directed avoids the use of other components. If tensions are historically high in the neck spherical appendix, it should be noted, however, that the facility is subject to breakage and, being the weakest of the whole, will be the first to succumb. In fact, although the tensions far less, the implant material (titanium grade 4) is less resistant than the material of the pillar (titanium grade 5) which has a breaking almost double. For the purposes of a more general analysis, the fact that the ball abutments are usually used in groups of 2, 3, or 4, and never alone, should however be considered. Also rarely they undergo purely bending loads or with strong angulation. The specifications in fact impose a maximum angle of 10° from the vertical axis and it is estimated that in any case the chewing loads can never reach angles close to 45°. In reality, then, this system, in a context of real application, with loads higher than normal and not very angled, should be fully responsive to the needs of resistance. For connections with pillars (studies A and B), it is still worth observing that the thread more generously combined with the fact of not having connecting screws makes it possible to avoid the weak parts, increasing in this way the security of the whole without detracting from the stability of the prosthesis [28–31].

Model B, formed by the implant system more pillar locator, is the system that appears to have characteristics of stress distribution better. It is resistant to all types of stress and has a yield strength in load of the system only to pure bending that, as already mentioned, is a type of extreme load that does not occur in reality. All the other tests show concentrations of stresses below the limits of the guard in all the components, allowing an optimum distribution of the voltages. Minimum values are noted even compared to other cases and for this it can be said that the connection is more convincing. The top coupling with the insert retention offers large surfaces that do not allow dangerous concentrations and the union on the implant through direct threading allows a good distribution of stresses on the whole system below. Even Model C, formed by the screw and implant abutments more, presents critical loads very angled similarly to study A. Model C has strong limitation related to the choice of analyzing just a regular universal abutment. The abutments used for dental implant overdentures have to be modelled according to several parameters like dental implant angulation, vertical dimension of the patient, and size of the overdenture. For this reason, Model C simply reflects the possibility of using the abutment like a standard dimension structure, screwed to the dental implant stressed by masticatory load.

For the principle of the lever arm on the masticatory loads, the very high upper part contributes to the increase of the voltages in the cases of load angle. Indeed, we have considered the universal pillar in its configuration crude, still not shaped according to the specific prosthesis that will be mounted above. The critical values are also due to excessive pressure on the implant, being built as we said with a relatively weaker, it ends up giving when loads exceed a certain value and angle. It is important to note the macroscopic differences of the three studies: in study A, maximum concentrations of tension occur in the neck of spherical appendix, whereas in studies B and C will have on the implant in the area of contact with the pillar. The results of the efforts calculated with finite elements, according to the theory of von Mises, have shown how large parts of the stresses are the prerogative of the pillars/abutment where it is in contact with the implant in the area at the border with the cortical bone. The material of these, however, is very resistant and at the end of the implant is at risk the most. To improve critical conditions of cases B and C, it would be enough to improve the implant material, using the titanium alloy even for the latter. It has already been described as the bone strength can vary depending on many factors: the specific area, the individual, his age, and so forth. The characteristics of the bone, both cortical and cancellous bone, were taken as the average from past studies only for global characterization systems. Also you do not have data for resistance to pure bending. Some studies give the same values of resistance in both tension and compression, and if so it was also assumed in our case that the values would be within the limits of safety. It may also take more general considerations. Apart from what is done in study A, and except for the concentrations of maximum stresses in contact between the pillars and the implant, it can be noted that a large distribution of the mechanical stresses occurs where the bone is in contact with the implant. Past studies have shown that when the maximum stress concentration occurs in the cortical bone, it is localized in the area of contact with the implant; when, instead, the maximum concentration of stress occurs in cancellous bone, it affects the apical area of the implant [26–33]. In this study, we can see exactly what has just been said, a sign that the tests were carried out in line with other tests carried out in the past. In cortical bone, the dissipation of the load is limited to the immediate area around the plant, while in the spongy bone stress distribution occurs in a much wider area. The cortical bone, having a modulus of higher elasticity, is stronger and more resistant to deformation; for that reason, it bears a greater load, in comparison to cancellous bone, in various clinical situations [34–39]. The connections of study B have proved valid choices from the biomechanical point of view: they guarantee a correct coupling and allow a good redistribution of the loads avoiding anomalous voltage spikes. We could say that if we consider the real loads, lower than those used in this discussion, and no extreme angles, all connections of abutment/implant designed allow a good margin of safety with the possibility of structural support of still physiological stress. As always, however, clinical trials are needed to verify the results obtained from the virtual model.

In conclusion, after the various simulations on the three systems/plants, analyzing the values of the maximum stresses generated by the five types of cargo, we can say that the system that best responds to extreme stress in question is study B. The locator abutment is therefore free and biomechanically more effective.

## Figures and Tables

**Table 1:** Mechanical properties of the materials used in the study.

Mechanical properties of the materials					
	Titanium grade 4	Titanium alloy Ti6Al4V	Cortical bone	Cancellous bone	Elitor
Density [kg/m^3^]	4510	4419	1800	1200	15000

Young's modulus (*E*) [Mpa]	105000	108000	9600 (*E*_*x*_)	144 (*E*_*x*_)	95000
			9600 (*E*_*y*_)	99 (*E*_*y*_)	
			17800 (*E*_*z*_)^*∗*^	344 (*E*_*z*_)^*∗*^	

Tangent elastic modulus (*G*) [Mpa]	—	—	3097 (*G*_*xy*_)	53 (*G*_*xy*_)	—
			3510 (*G*_*yz*_)	63 (*G*_*yz*_)	
			3510 (*G*_*xz*_)	45 (*G*_*xz*_)	

Poisson's ratio (*ν*)	0,37	0,37	0,55 (*ν*_*xy*_)	0,23 (*ν*_*xy*_)	0,35
			0,3 (*ν*_*yz*_)	0,11 (*ν*_*yz*_)	
			0,3 (*ν*_*xz*_)	0,13 (*ν*_*xz*_)	

Tensile yield strength (*σ*_*y*_) [Mpa]	485	830	115	32,4	700

Tensile ultimate strength (*σ*_*u*_)	550	900	133	37,5	855

Compressive yield strength (*σ*_*y*_) [Mpa]	485	830	182	51	700

Compressive ultimate strength (*σ*_*u*_) [Mpa]	—	—	195	55	—

*∗*: maximus value of stress.

**Table 2:** Maximum elastic deformation and the maximum tensions (Model A).

COMP.	Equiv. elastic strain (max)	0,028596 mm/mm	Midollar bone
	Equivalent stress (max)	499,88 MPa	Neck of the implant

C. 45°	Equiv. elastic strain (max)	0,038897 mm/mm	Neck of the implant
	Equivalent stress (max)	**4180,8 MPa**	Neck of the implant

F. 90°	Equiv. elastic strain (max)	0,048162 mm/mm	Neck of the implant
	Equivalent stress (max)	**5186,8 MPa**	Neck of the implant

T. 45°	Equiv. elastic strain (max)	0,039314 mm/mm	Neck of the implant
	Equivalent stress (max)	**4234,1 MPa**	Neck of the implant

TRAZ.	Equiv. elastic strain (max)	0,028613 mm/mm	Midollar bone
	Equivalent stress (max)	499,89 MPa	Neck of the implant

**Table 3:** Maximum stresses for component [MPa] (Model A).

Load	Insert	Sheath	Abutment	Implant	Cortical bone	Midollar bone
COMP.	98,62	150,72	499,88	176,58	64,22	4,91
C. 45°	271,34	346,86	**4180,8**	**552,93**	142,68	7,73
F. 90°	345,02	483,04	**5186,8**	**633,07**	*161,17*	9,16
T. 45°	258,55	333,57	**4234,1**	**569,81**	**157,47**	7,68
TRAZ.	71,09	152,57	499,89	177,12	65,17	4,95

**Table 4:** Maximum elastic deformation and the maximum tensions (Model B).

COMP.	Equiv. elastic strain (max)	0,027808 mm/mm	Midollar bone
	Equivalent stress (max)	197,73 MPa	Abutment implant

C. 45°	Equiv. elastic strain (max)	0,031117 mm/mm	Midollar bone
	Equivalent stress (max)	443,29 MPa	Abutment implant

N. 90°	Equiv. elastic strain (max)	0,031577 mm/mm	Midollar bone
	Equivalent stress (max)	*487,85 MPa*	Abutment implant

T. 45°	Equiv. elastic strain (max)	0,031237 mm/mm	Midollar bone
	Equivalent stress (max)	453,52 MPa	Abutment implant

TRAZ.	Equiv. elastic strain (max)	0,027824 mm/mm	Midollar bone
	Equivalent stress (max)	197,14 MPa	Abutment implant

**Table 5:** Maximum stresses for component [MPa] (Model B).

Load	Insert	Abutment	Implant	Cortical bone	Midollar bone
COMP.	167,21	160,52	197,73	67,94	4,75
C. 45°	241,62	318,03	443,29	150,07	6,58
F. 90°	259,21	382,32	*487,85*	*154,8*	7,11
T. 45°	217,42	377,75	453,52	**156,1**	6,29
TRAZ.	167,24	159,59	197,14	67,94	4,75

**Table 6:** Maximum stresses for component [MPa] (Model C).

COMP.	Equiv. elastic strain (max)	0,027505 mm/mm	Midollar bone
	Equivalent stress (max)	206,47 MPa	Abutment implant

C. 45°	Equiv. elastic strain (max)	0,040865 mm/mm	Midollar bone
	Equivalent stress (max)	**635,08 MPa**	Abutment implant

N. 90°	Equiv. elastic strain (max)	0,047132 mm/mm	Midollar bone
	Equivalent stress (max)	**856,52 MPa**	Abutment implant

T. 45°	Equiv. elastic strain (max)	0,041481 mm/mm	Midollar bone
	Equivalent stress (max)	**732,56 MPa**	Abutment implant

TRAZ.	Equiv. elastic strain (max)	0,027519 mm/mm	Midollar bone
	Equivalent stress (max)	195,09 MPa	Cortical bone

**Table 7:** Maximum stresses for component [MPa] (Model C).

Load	Abutment	Passing screw	Implant	Cortical bone	Midollar bone
COMP.	158,57	103,69	206,47	66,62	4,82
C. 45°	559,35	220,43	**635,08**	175,88	8,55
F. 90°	672,61	283,67	**856,52**	**210,91**	10,79
T. 45°	550,2	224,38	**732,56**	**189,13**	8,61
TRAZ.	147,85	100,58	195,09	65,05	5
